# Prevalence of Breast Cancer-Related Lymphedema in Israeli Women Following Axillary Procedures

**DOI:** 10.3390/jcm14030688

**Published:** 2025-01-22

**Authors:** Daniel Josef Kedar, Elad Zvi, Oriana Haran, Lior Sherker, Michael Sernitski, Nadav Oppenheim, Eran Nizri, Marian Khatib, Yoav Barnea

**Affiliations:** 1 Department of Plastic and Reconstructive Surgery, Tel Aviv Sourasky Medical Center, Affiliated to the Faculty of Medicine, Tel Aviv University, Tel Aviv 6997801, Israelybarnea@gmail.com (Y.B.); 2 Peritoneal Surface Malignancy and Melanoma Unit, Department of Surgery B Division, Tel Aviv Sourasky Medical Center, Affiliated to the Faculty of Medicine, Tel Aviv University, Tel Aviv 6997801, Israel; 3 Breast Surgery Unit, Department of Surgery, Tel Aviv Sourasky Medical Center, Affiliated to the Faculty of Medicine, Tel Aviv University, Tel Aviv 6997801, Israel

**Keywords:** breast cancer-related lymphedema, Israel, patient-reported outcomes

## Abstract

**Background/Objectives:** Breast cancer-related lymphedema (BCRL) is a chronic complication of breast cancer treatment, leading to physical and psychological morbidity. While widely studied globally, the prevalence and risk factors for BCRL in Israeli patients remain unexplored. This study’s objectives were to determine the prevalence of BCRL in Israeli women treated for breast cancer, validate the Hebrew-translated Norman Questionnaire (NQ) for BCRL screening, and identify risk factors associated with the condition. **Methods:** A single-center study was conducted at Tel Aviv Sourasky Medical Center, including 181 patients who underwent unilateral axillary lymph node interventions between 2015 and 2018. Participants completed the Hebrew-translated NQ, which was validated through clinical evaluation and circumference-based volume measurements in a subset of 20 patients. Prevalence rates and risk factors were analyzed using multivariate modeling. **Results:** The prevalence of BCRL was 20%, with rates varying by procedure: 8.9% for sentinel lymph node biopsy, 19.6% for lymph node sampling, and 37.5% for axillary lymph node dissection (ALND). Of the 35 patients with BCRL, only 14% had been previously diagnosed. Risk factors included ALND (OR = 97.31), a higher lymph node excision count (OR = 0.81), and referral to physiotherapy (OR = 133.50). The Hebrew NQ demonstrated strong validity (rs = 0.852; *p* < 0.001). **Conclusions:** This is the first study to estimate BCRL prevalence in Israeli women, highlighting underdiagnosis and the need for improved early detection. The Hebrew NQ is a reliable screening tool, enabling timely referral and intervention. Early diagnosis is crucial for optimizing treatment outcomes and improving the quality of life of BCRL patients.

## 1. Introduction

Lymphedema is a chronic, progressive, and often debilitating condition that arises from the dysfunction of the lymphatic system. It is characterized by the abnormal accumulation of protein-rich fluid in the interstitial spaces, leading to inflammation, hypertrophy of adipose tissue, and fibrosis. This process results in swelling, stiffness, and eventual deformity in the affected region, which can severely impair mobility, functionality, and quality of life. Over time, the chronic nature of lymphedema not only causes physical discomfort but also imposes a profound psychological burden, including anxiety, depression, and social withdrawal for patients who struggle with the condition [1].

While lymphedema has been documented in medical literature for centuries, it has garnered increased attention in recent decades, particularly due to its emergence as a frequent complication of cancer treatments, including breast cancer therapies. As survival rates for breast cancer continue to improve globally, the focus has shifted toward addressing the long-term consequences of cancer treatment, with breast cancer-related lymphedema (BCRL) standing out as a major concern.

Lymphedema is generally categorized into two distinct types based on its etiology: primary lymphedema and secondary lymphedema. Primary lymphedema, which is less common, is caused by congenital abnormalities, genetic mutations, or vascular malformations that impair lymphatic system development or function. Clinical manifestations of primary lymphedema can occur at various stages of life: at birth (10–25% of cases), before the age of 35 (70% of cases), or even later in adulthood (10%) [2,3].

Secondary lymphedema, in contrast, is far more prevalent and arises due to external factors that disrupt or damage the lymphatic system. These factors include infections, trauma, surgeries, or medical interventions, such as radiation therapy. Among secondary lymphedema cases, breast cancer-related lymphedema (BCRL) is particularly significant. BCRL is primarily a result of surgical lymph node dissection, sentinel lymph node biopsy (SLNB), and/or radiation therapy to the axillary region during breast cancer treatment. Clinically, patients with BCRL commonly experience a spectrum of symptoms, including tension, heaviness, swelling, pain, and reduced limb function. Over time, if left untreated, these symptoms worsen due to fat deposition and fibrosis, leading to chronic impairments that dramatically reduce the patient’s quality of life. Psychological consequences are also well-documented, with an increased prevalence of depression and anxiety among affected individuals [4].

In Israel, breast cancer is the most common malignancy among women, with an incidence rate of approximately 101 cases per 100,000 Jewish women and 66 cases per 100,000 Arab women. On average, around 4500 new breast cancer cases are diagnosed in Israel each year [5]. Improved awareness, early detection through screening programs, and advancements in treatment protocols have significantly increased survival rates. However, with this increased survivorship comes the emergence of long-term treatment-related complications, such as BCRL, which remain under-recognized and under-researched in the Israeli population. Despite its clinical and psychological impact, there are currently no published studies examining the prevalence of BCRL among Israeli breast cancer survivors.

Volume measurements are commonly used to diagnose and evaluate BCRL [6]. One notable tool is the Norman Questionnaire (NQ), a validated self-reported diagnostic questionnaire developed by Norman et al. [7]. It uses simple questions to detect BCRL by comparing limb size and identifying related symptoms. The NQ has shown high sensitivity (0.86–0.92) and specificity (0.90) and has been translated and validated in multiple languages [8].

## 2. Methods

This study aimed to evaluate the prevalence of BCRL among Israeli breast cancer patients while simultaneously validating the Hebrew version of the Norman Questionnaire (NQ). This study was conducted between July 2022 and August 2023 at the Tel Aviv Sourasky Medical Center (Ichilov) and received approval from the local Ethics Committee (reference: 0935-20-TLV, approved on 28 February 2022).

The English NQ was translated into Hebrew following international guidelines [9]. Eligible participants included women over age 18 who underwent unilateral axillary lymph node procedures (sentinel lymph node biopsy [SLNB], sampling, or lymph node dissection) for breast cancer between January 2015 and December 2018. The data collected included demographics, cancer details, surgical outcomes, and post-surgical treatments.

A total of 342 patients met the inclusion criteria, 181 patients consented to participate, and we were able to complete all the data on 174 patients.

The data collection involved three main components:

- Self-reported assessments: Participants completed the Hebrew NQ questionnaire. For reliability testing, the questionnaire was administered twice: once over the phone or within 24 h prior to a hospital visit and again during their hospital appointment under the supervision of the research team. This method allowed for a comprehensive evaluation of participants’ perspectives and experiences and involved two stages of questionnaire completion, both at home and during the hospital visit. Furthermore, the research team’s presence ensured clarity and consistency in the responses, while additional measurements provided objective data to complement the self-reported information.
- Clinical measurements: To complement the self-reported data, objective arm volume measurements were conducted using a retractable, non-stretch tape measure on a subset of 20 patients (10 positive and 10 negative for BCRL based on the NQ). Measurements were taken at fixed intervals (7, 14, 21, and 28 cm) both above and below the olecranon, with the proximal edge serving as the reference point. Limb volumes were calculated using the frustum of a cone formula. A correction factor was applied to account for natural volume differences between dominant and non-dominant arms [10]. Finally, the relative excess arm volume was determined by the following formula: (absolute difference volumes between both arms/(V_1_ swollen limb + V_2_ unaffected limb)/2) × 100. As in past research on healthy subjects, a mean difference of 3.2% between the right and left arms was found, and a correction factor was applied to adjust the volume of the non-dominant arm [10].
- Data collection from patients’ medical records: This study included breast cancer patients who underwent one of the following axillary interventions: sentinel lymph node biopsy (SLNB), lymph node sampling, or axillary lymph node dissection (ALND). These represent a spectrum of procedures with varying degrees of invasiveness. SLNB involves identifying and removing only the first few lymph nodes (sentinel nodes) to which cancer is likely to spread, minimizing complications, and preserving lymphatic function. ALND is the most extensive procedure, involving the removal of multiple lymph nodes from the axilla to address advanced disease or confirm staging, but it carries a higher risk of complications, such as lymphedema. Any procedure considered by the surgeons as more than an SLNB and less extensive than a proper ALND was classified as lymph node sampling [11].

## 3. Results

The results of this study demonstrate the reliability and validity of the Hebrew NQ for detecting BCRL among Israeli breast cancer patients.

For the validation process of the Hebrew NQ, a comparison of the limbs’ volume between the 10 positive and 10 negative for BCRL based on the questionnaire was conducted using the Wilcoxon signed-rank test. None of these patients had a prior diagnosis of lymphedema.

**Figure 1:**
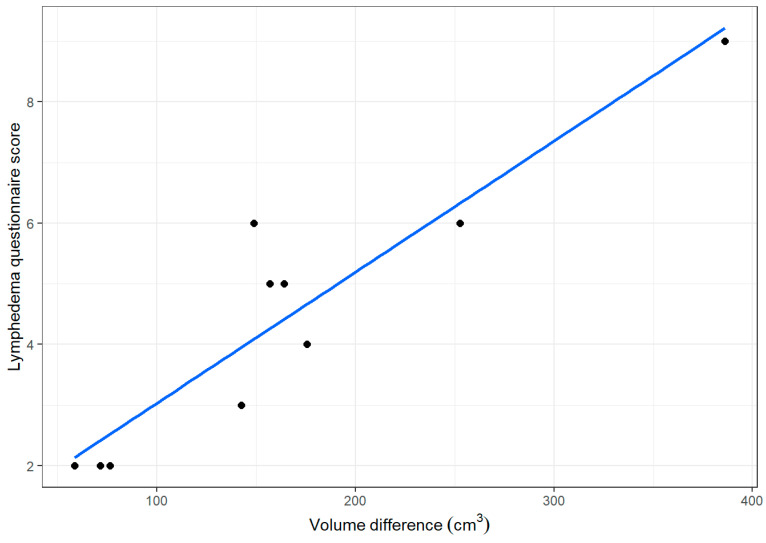
Correlation between the questionnaire score and the physical exam.

For the non-lymphedema group, no significant difference in the limbs’ volume was found (*p* = 0.19). In the group of patients with lymphedema, the edematous limb (median = 1721.35 cm^3^) showed higher values (*p* < 0.01) of volume compared with the non-edematous group (median = 1597.6 cm^3^). In addition, Spearman’s correlation between the questionnaire score and the difference between edematous and non-edematous limbs were computed. The resulting correlation indicated a strong positive linear relation between the two quantities (r_s = 0.852, *p* < 0.001) (Figure 1).

In our study, data were collected from 181 patients. Overall, the patient population in this study presented a diverse group in terms of age, BMI, and cancer type. The age range was broad, from 26 to 85 years, and the mean age across the groups was 56.1 years. The overall mean BMI was 26.0, ranging from 18.3 to 43.3. Most patients had intraductal carcinoma. Furthermore, 79% underwent breast conservative surgery, and 21% underwent mastectomy. In total, 62 of the patients (34.25%) underwent breast reconstruction, of which 72.6% (45) were reconstructed with breast implants, and 27.4% (17) underwent autologous reconstruction. Moreover, 10.5% of the patients were positive for the BRCA 1 or BRCA 2 gene. There was a notable difference in the rates of neoadjuvant treatment, with 64.1% of the overall group receiving it. The ALND group had the lowest percentage (43.6) with a statistically significant difference between the groups. The mean number of lymph nodes removed varied significantly among the groups. The SLNB and sampling groups averaged around 3.5 nodes, while the ALND group averaged 11.3 nodes, with a statistically significant difference (Table 1).

**Figure 2:**
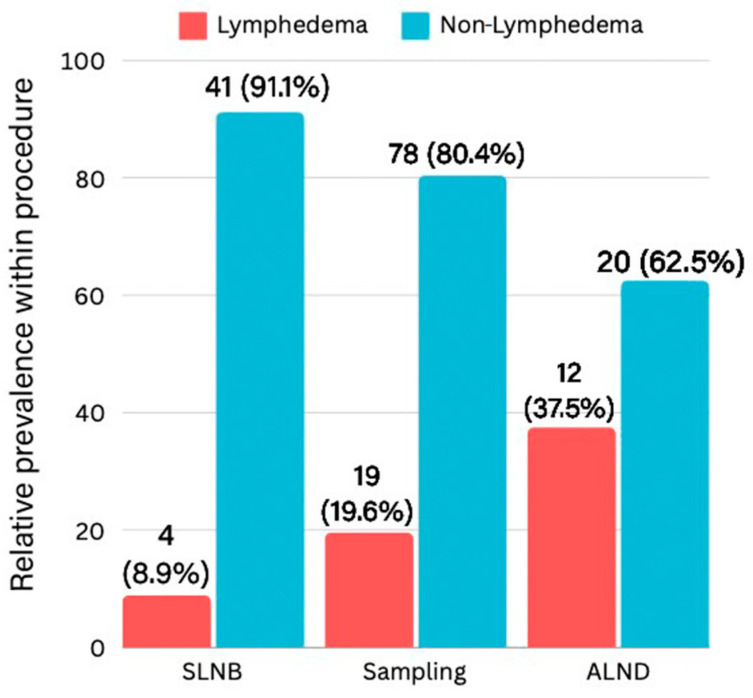
The prevalence of lymphedema across the different axillary procedures. Sentinel lymph node biopsy (SLNB): 8.9% (4/45); lymph node sampling: 19.6% (19/97); axillary lymph node dissection (ALND): 37.5% (12/32).

Of the 174 patients for whom complete data were available, 45 patients underwent sentinel lymph node biopsy, 97 patients underwent lymph node sampling, and 32 patients underwent proper axillary lymph node dissection. Out of the 174 participants, 20% (*n* = 35) were diagnosed with BCRL based on the Hebrew NQ. The prevalence rates varied by the procedure type (Figure 2).

In order to determine how the procedure type affected the risk of suffering from lymphedema, we fit a baseline model with three predictors (for the three different procedures) and then performed a stepwise model selection method based on the AIC values to identify a suboptimal model. The total number of predictors included was 25, with the following factors: BMI, age, smoking, medical history (hypertension, hypothyroidism, diabetes mellitus, IHD, peripheral vascular disease, and hypercoagulability), type of neoadjuvant treatment, adjuvant radiation treatment, existence of the BRCA gene, number of lymph nodes involved with tumor, number of lymph nodes resected, type of breast reconstruction, and complications, such as infection, wound dehiscence, and hematoma or seroma formation.

Risk factor analysis revealed that ALND and referral to physiotherapy were associated with an increased risk of BCRL. We also found that the lower the number of lymph nodes excised, the lower the risk for the development of lymphedema that was noted. Table 2 presents the predictors identified through stepwise model selection.

## 4. Discussion

The translation of the Norman Questionnaire (NQ) into Hebrew aimed to provide a reliable and valid tool for the detection and characterization of breast cancer-related lymphedema (BCRL) in Hebrew-speaking populations. The Hebrew NQ demonstrated strong reliability and validity, aligning with findings from its original and translated versions.

As part of the validation process for the Hebrew NQ, 10 patients who reported a positive response to the “difference in size” symptom in the questionnaire but had no prior lymphedema diagnosis underwent clinical evaluation. Circumference measurements revealed a significant difference between the affected and healthy limbs in all 10 patients, compared with no significant differences in 10 patients with negative ratings.

However, the diagnostic criteria for lymphedema vary significantly, affecting prevalence estimates. The commonly accepted thresholds include an absolute interlimb difference of a >2 cm circumference or a >200 mL volume, while others suggest a >3% volume increase, with more conservative definitions requiring a >10% volume difference. Using these criteria, all 10 positively rated patients met the >2 cm circumference threshold, while only 2 had >200 mL volume differences. All 10 fit the >3% volume increase criterion, but only 4 met the >10% difference threshold. These findings underscore the variability in lymphedema definitions, which contribute to reported incidence rates ranging from <5% to >50% [12].

This is the first study investigating the prevalence of BCRL in Israeli breast cancer patients. The 20% prevalence observed correlates with global meta-analyses reporting rates of 21.4% [10]. These findings show that lymphedema is a common complication for breast cancer survivors and more emphasis should be put on the diagnosis and treatment of lymphedema.

Moreover, this study revealed that only 14% (5/35) of BCRL cases were previously diagnosed, highlighting low awareness and significant underdiagnosis in Israel. Early detection is crucial, as timely interventions can prevent progression and improve patient outcomes. Early diagnosis is critical to prevent progression to fibrosis and adipose deposition, which complicates treatment. The NQ provides a simple screening tool to identify patients needing further evaluation.

Risk factors such as ALND and lymph node removal are consistent with previous research [13]. However, unlike other studies, we did not identify radiation therapy or BMI as significant risk factors, highlighting potential differences in our population or methodology.

Subclinical edema following surgery has been identified as a significant risk factor for the subsequent development of lymphedema, emphasizing the importance of early monitoring for limb swelling to identify at-risk patients [6]. This aligns with our study’s findings, which suggest a higher prevalence of lymphedema among patients who were referred early to physiotherapy.

Historically, BCRL was treated only when the patient or caregiver identified visible swelling in the limbs. However, advancements in the understanding and treatment of BCRL have shifted the focus toward early detection, proactive monitoring, and identifying at-risk groups, as early intervention is associated with significantly better outcomes.

In our study population, patients were not routinely referred to breast rehabilitation, with only 33 patients receiving physiotherapy referrals. Notably, most of these referrals were made only after the onset of significant symptoms. These findings underscore the need for greater emphasis on early detection and proactive intervention to address breast cancer-related lymphedema (BCRL) before symptoms progress and become more challenging to manage [14].

The prevalence of BRCA gene mutations in the general population is estimated to be 0.2–0.3%, with a significantly higher incidence of 2–2.5% among Ashkenazi Jews. Approximately 12% of breast cancers in the Ashkenazi Jewish population are attributable to mutations in the BRCA1 or BRCA2 gene. In our study, the incidence of BRCA mutations was notably high at 10.5%, which may be explained by the fact that most patients in our cohort were Ashkenazi Jews. Additionally, our hospital houses a dedicated center for the follow-up and treatment of BRCA gene carriers, likely leading to an over-representation of this population in our study [15].

The validated Hebrew Norman Questionnaire (NQ) offers significant potential for integration into routine clinical practice as a practical and reliable tool for the early detection of breast cancer-related lymphedema (BCRL). Its simplicity, cost-effectiveness, and ease of administration make it well-suited for use in both hospital settings and community-based care. By identifying patients at risk of or already experiencing lymphedema, the NQ enables clinicians to intervene early, potentially preventing the progression of symptoms and associated complications. Furthermore, its use in regular follow-up appointments for breast cancer survivors could enhance surveillance and improve outcomes by facilitating timely referrals for physiotherapy or other therapeutic interventions. Given the high sensitivity and specificity demonstrated in our study, integrating the NQ into clinical workflows could significantly improve the management of BCRL while reducing the burden of underdiagnosis. Future research should explore its implementation on a larger scale and assess its impact on long-term patient outcomes.

Improved diagnostic technologies now allow for the detection of subtle changes in limb volume at early stages, enabling timely referrals for lymphatic physiotherapy or surgical interventions [16].

Lymphoscintigraphy remains the gold-standard imaging modality for assessing lymphatic pathways. This technique involves injecting a technetium-99-labeled tracer into the dermis at the limb’s extremity to evaluate dynamic responses, areas of obstruction, and retrograde lymphatic flow [17]. Magnetic resonance lymphangiography (MRL) has also been explored, though it is not widely adopted [18]. In contrast, indocyanine green (ICG) lymphography has emerged as a promising diagnostic and prognostic tool, offering the advantage of avoiding radioactive tracers. These imaging methods facilitate personalized treatment planning for BCRL patients, encompassing both conservative and surgical therapies [19].

**Figure 3:**
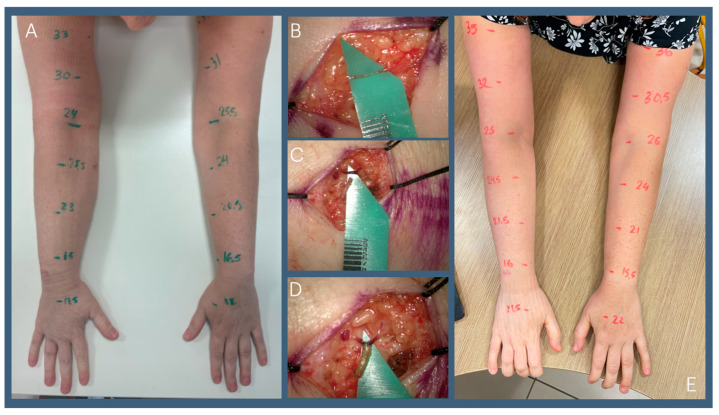
Forty-two-year-old patient who suffers from Rt BCRL. (**A**) Pre-op; (**B**) 0.3 mm lymphatic vessel in the distal arm; (**C**) 0.6 mm vein was found (pre-operative ultrasound assisted in locating lymph vessel and adjacent vein); (**D**) side-to-end anastomosis of lymph vessel to vein using 11-00 nylon sutures; (**E**) 3-months post-op shows improvement in limb circumferences.

**Figure 4:**
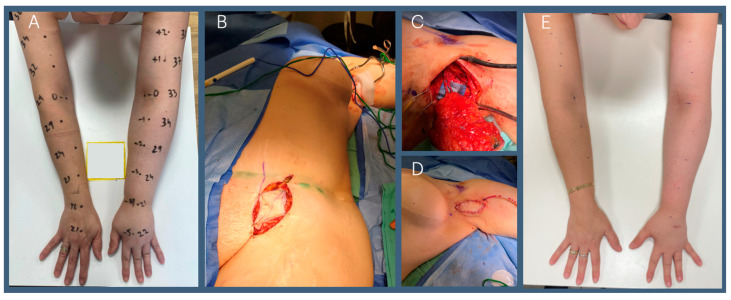
Forty-nine-year-old patient with Lt BCRL. (**A**) Pre-op; (**B**) vascularized lymph node harvest from left groin; (**C**) following scar removal, the flap was transferred to the Lt axilla; (**D**) a small skin island was left as a way to monitor the flap and its viability; (**E**) 6-months post-op with improvement in limb circumferences.

The surgical management of lymphedema includes physiological and reductive approaches. Physiological methods such as lymphaticovenous bypass (LVB) and vascularized lymph node transfer (VLNT) aim to restore lymphatic circulation. LVB connects high-pressure lymphatic vessels to low-pressure venules, improving fluid drainage (Figure 3), while VLNT transplants functional lymph nodes to re-establish lymphatic flow (Figure 4). Both techniques are most effective when performed early, before the development of lymphatic sclerosis, fibrosis, or excess fat deposition [20,21,22], underscoring the critical need for early diagnosis and intervention.

The generalizability of this study’s findings on breast cancer-related lymphedema (BCRL) and the validation of the Hebrew Norman Questionnaire (NQ) to other populations warrants careful consideration. Variations in genetic, demographic, and healthcare system factors across countries may influence the prevalence, detection, and management of BCRL. For example, differences in genetic predispositions, such as BRCA mutations, and access to diagnostic tools, like lymphoscintigraphy or ICG lymphography, may impact the feasibility of replicating these findings globally. Furthermore, socioeconomic and cultural factors, including healthcare access, patient awareness, and attitudes toward post-cancer care, could affect the adoption of novel screening tools and interventions. To ensure broader applicability, the NQ should be validated in other languages and cultural contexts, with attention to maintaining its high sensitivity and specificity. Future multinational studies are essential to evaluate the efficacy of these approaches in diverse populations and to identify region-specific risk factors, healthcare barriers, and best practices for the early detection and management of BCRL.

This study had several limitations that should be considered when interpreting its findings. First, the sample size, while sufficient for initial validation of the Hebrew Norman Questionnaire (NQ), may limit the generalizability of the results to the broader Israeli breast cancer survivor population. Additionally, this study was conducted at a single medical center, which may have introduced selection bias and may limit its applicability to other healthcare settings. The reliance on self-reported data for assessing BCRL could also have introduced reporting bias, despite efforts to complement this with objective limb volume measurements. Another limitation was the lack of long-term follow-up to assess the progression and outcomes of BCRL over time, particularly for patients who were not referred to early rehabilitation. Finally, differences in the diagnostic criteria for lymphedema across studies highlight the need for standardized thresholds, which may affect comparisons with other research. Future studies should address these limitations through multicenter designs, larger and more diverse samples, and longitudinal assessments to strengthen the validity and applicability of the findings.

## 5. Conclusions

In the era of de-escalation surgery, cases of BCRL have shown a decreasing trend, largely due to the adoption of less-invasive techniques, such as sentinel lymph node biopsy (SLNB) over axillary lymph node dissection (ALND), and advancements in radiation therapy. However, despite these advancements, lymphedema remains a major complication and a prevalent issue among breast cancer survivors. This study highlights breast cancer-related lymphedema as a significant complication affecting approximately 20% of Israeli breast cancer survivors, underscoring the ongoing clinical and psychological burden of this condition. These findings highlight the need for continued emphasis on early detection, effective management, and further refinement of treatment approaches to minimize the impact of lymphedema, particularly in high-risk populations. The validated Hebrew Norman Questionnaire provides a practical and reliable screening tool to enable early detection and timely intervention. As survivorship improves, addressing complications like BCRL must become a priority to enhance long-term outcomes and quality of life for breast cancer patients in Israel.

## Figures and Tables

**Table 1:** Comparative Demographic, Clinical, and Treatment Characteristics in Breast Cancer Patients following axillary interventions.

	SLNB (N = 44)	Sampling/Sentinel (N = 98)	ALND (N = 39)	Overall (N = 181)	*p*-Value
**BMI**					
Mean (SD)	25.7 (5.09)	25.4 (4.64)	27.8 (5.49)	26.0 (5.00)	0.068
Median [min., max.]	24.5 [18.3, 40.0]	24.2 [18.4, 40.0]	25.5 [19.4, 43.3]	24.6 [18.3, 43.3]	
Missing	0 (0%)	1 (1.0%)	0 (0%)	1 (0.5%)	
**Age at surgery**					
Mean (SD)	57.2 (13.3)	55.8 (12.1)	55.6 (15.1)	56.1 (13.0)	0.816
Median [min., max.]	59.0 [26.0, 79.0]	57.0 [27.0, 85.0]	51.0 [31.0, 83.0]	56.5 [26.0, 85.0]	
**Type of cancer**					
Intraductal carcinoma	39 (88.6%)	85 (86.7%)	29 (74.4%)	153 (84.5%)	0.415
Intralobular carcinoma	3 (6.8%)	10 (10.2%)	8 (20.5%)	21 (11.6%)	
Mucinous carcinoma	2 (4.5%)	3 (3.1%)	2 (5.1%)	7 (3.9%)	
**BRCA 1/2 gene**					
Negative	41 (93.2%)	82 (83.6%)	36 (92.3%)	159 (87.8%)	0.2
Positive	2 (4.5%)	14 (14.3%)	3 (7.7%)	19 (10.5%)	
Missing	1 (2.3%)	2 (2.1%)	0 (0%)	3 (1.7%)	
**Surgery type**					
Lumpectomy	34 (77.3%)	82 (83.7%)	27 (69.2%)	143 (79.0%)	0.147
Mastectomy	10 (22.7%)	16 (16.3%)	12 (30.8%)	38 (21.0%)	
**Neoadjuvant treatment**					
Yes	30 (68.2%)	69 (70.4%)	17 (43.6%)	116 (64.1%)	0.0158
No	13 (29.5%)	29 (29.6%)	21 (53.8%)	63 (34.8%)	
Missing	1 (2.3%)	0 (0%)	1 (2.6%)	2 (1.1%)	
**Adjuvant radiation therapy**					
No	12 (27.3%)	16 (16.3%)	4 (10.3%)	32 (17.6%)	0.12
Yes	32 (72.7%)	81 (82.6%)	35 (89.7%)	148 (81.7%)	
Missing	0 (0%)	1 (1.1%)	0 (0%)	1 (0.7%)	
**Number of lymph nodes removed**					
Mean (SD)	3.52 (2.50)	3.43 (2.54)	11.3 (5.67)	5.16 (4.72)	<0.001
Median [min., max.]	3.00 [1.00, 10.0]	3.00 [1.00, 19.0]	11.0 [2.00, 26.0]	3.00 [1.00, 26.0]	
Missing	0 (0%)	3 (3.0%)	0 (0%)	3 (1.6%)	

**Table 2:** Stepwise model selection method based on Akaike Information Criterion values for identifying risk factors for the development of breast cancer-related lymphedema.

	Questionnaire Score		
Predictor	Odds Ratio	CI	*p*-Value
(Intercept)	0.00	0.00–0.08	**0.005**
Adjuvant radiation	3.99	0.70–32.96	0.154
BMI	1.11	0.97–1.28	0.128
BRCA-positive	0.24	0.03–1.46	0.152
Hypertension	0.25	0.04–1.03	0.076
Lymph nodes involved at time of diagnosis	0.18	0.02–1.12	0.085
Lymphedema diagnosis	17.43	1.18–399.34	**0.047**
SLNB	Reference		
Sampling/sentinel	5.62	0.94–54.01	0.090
ALND	97.31	6.42–3117.21	**0.003**
No. of lymph N. removed	0.81	0.66–0.95	**0.021**
Physiotherapy	133.50	25.75–1311.61	**<0.001**
Observations	156		
R^2^ Tjur	0.584		

The bold are the ones that are statistically significant.

## Data Availability

The data will be made available upon request to the corresponding author.
